# Prenatal detection of congenital heart defects using the deep learning-based image and video analysis: protocol for Clinical Artificial Intelligence in Fetal Echocardiography (CAIFE), an international multicentre multidisciplinary study

**DOI:** 10.1136/bmjopen-2025-101263

**Published:** 2025-06-05

**Authors:** Olga Patey, Netzahualcoyotl Hernandez-Cruz, Elena D’Alberti, Bojana Salovic, J Alison Noble, Aris T Papageorghiou, CAIFE Research Group, Theophilus Adu-Bredu

**Affiliations:** 1 Nuffield Department of Women’s & Reproductive Health, University of Oxford, Oxford, England, UK; 2 Brompton Fetal Centre, Paediatric Cardiology, Royal Brompton Hospital, London, England, UK; 3 Fetal Medicine Unit, St George’s University Hospitals NHS Foundation Trust, London, England, UK; 4 Institute of Biomedical Engineering (IBME), University of Oxford, Oxford, England, UK; 5 St George’s University Hospitals NHS Foundation Trust, London, England, UK

**Keywords:** Artificial Intelligence, Congenital heart disease, Echocardiography, Diagnostic Imaging, Prenatal diagnosis, Pregnant Women

## Abstract

**Abstract:**

**Introduction:**

Congenital heart defect (CHD) is a significant, rapidly emerging global problem in child health and a leading cause of neonatal and childhood death. Prenatal detection of CHDs with the help of ultrasound allows better perinatal management of such pregnancies, leading to reduced neonatal mortality, morbidity and developmental complications. However, there is a wide variation in reported fetal heart problem detection rates from 34% to 85%, with some low- and middle-income countries detecting as low as 9.3% of cases before birth. Research has shown that deep learning-based or more general artificial intelligence (AI) models can support the detection of fetal CHDs more rapidly than humans performing ultrasound scan. Progress in this AI-based research depends on the availability of large, well-curated and diverse data of ultrasound images and videos of normal and abnormal fetal hearts. Currently, CHD detection based on AI models is not accurate enough for practical clinical use, in part due to the lack of ultrasound data available for machine learning as CHDs are rare and heterogeneous, the retrospective nature of published studies, the lack of multicentre and multidisciplinary collaboration, and utilisation of mostly standard planes still images of the fetal heart for AI models. Our aim is to develop AI models that could support clinicians in detecting fetal CHDs in real time, particularly in nonspecialist or low-resource settings where fetal echocardiography expertise is not readily available.

**Methods and analysis:**

We have designed the Clinical Artificial Intelligence Fetal Echocardiography (CAIFE) study as an international multicentre multidisciplinary collaboration led by a clinical and an engineering team at the University of Oxford. This study involves five multicountry hospital sites for data collection (Oxford, UK (n=1), London, UK (n=3) and Southport, Australia (n=1)). We plan to curate 14 000 retrospective ultrasound scans of fetuses with normal hearts (n=13 000) and fetuses with CHDs (n=1000), as well as 2400 prospective ultrasound cardiac scans, including the proposed research-specific CAIFE 10 s video sweeps, from fetuses with normal hearts (n=2000) and fetuses diagnosed with major CHDs (n=400). This gives a total of 16 400 retrospective and prospective ultrasound scans from the participating hospital sites. We will build, train and validate computational models capable of differentiating between normal fetal hearts and those diagnosed with CHDs and recognise specific types of CHDs. Data will be analysed using statistical metrics, namely, sensitivity, specificity and accuracy, which include calculating positive and negative predictive values for each outcome, compared with manual assessment.

**Ethics and dissemination:**

We will disseminate the findings through regional, national and international conferences and through peer-reviewed journals. The study was approved by the Health Research Authority, Care Research Wales and the Research Ethics Committee (Ref: 23/EM/0023; IRAS Project ID: 317510) on 8 March 2023. All collaborating hospitals have obtained the local trust research and development approvals.

**STRENGTH AND LIMITATIONS OF THE STUDY**

- The strength of the Clinical Artificial Intelligence Fetal Echocardiography (CAIFE) study is the project’s design as a multidisciplinary multicentral international study that will allow clinical and engineering teams to collaborate and collect large fetal echocardiographic datasets from various and diverse hospital populations.
- The novelty of the CAIFE project is the prospective data collection of the CAIFE sweeps, which ensure that artificial intelligence (AI) models are trained and evaluated according to the sequential segmental analysis of the fetal heart for complex congenital heart defect (CHD) diagnosis.^27^
- Although automated fetal echocardiography via AI-based analysis might improve the prenatal detection of CHDs, particularly in low- and middle-income countries, such applications are yet to be proven, requiring further research and development.

## Introduction

 Congenital heart defects (CHDs) represent structural and functional abnormalities of the heart presented at birth and are a significant and rapidly emerging global challenge in child health due to a global increase in the proportion of neonatal deaths due to CHDs.^1^ Affecting approximately 0.8% to 1.2% of live births worldwide, CHD is the most common congenital anomaly and a leading cause of neonatal and childhood mortality.^2^ Of the 1.5 million children born with CHD each year, 96% live in low- and middle-income countries (LMICs).^1 3^ Infant mortality rates associated with CHD are highest in LMICs and have increased over the past decades.^1^,^5^

Each year about a quarter of infants born with major CHD require surgery or intervention in the first year of life.^4^ Without prenatal diagnosis, these infants are less likely to survive to the point of surgery, less likely to survive after surgery^6 7^ and at greater risk of adverse neurological complications.^8 9^ This is because prenatal detection of CHDs enables early identification of fetal extracardiac abnormalities, facilitating the development of prenatal and postnatal management plans through multidisciplinary collaboration^9^; it also gives time for parents to make informed decisions on invasive prenatal diagnosis or pregnancy continuation. However, prenatal detection for CHDs is highly dependent on human skills and experience^10^; despite the evident importance of detection, in most cases, CHD is missed during basic anatomical screening.^11^

In research settings, the application of artificial intelligence (AI) (including deep learning-based) algorithms in fetal echocardiography has shown favourable results^12^,^23^ with detection rates of approximately 85% for fetal CHDs.^15^ However, several limitations persist in previous studies, including small ultrasound databases (due to the rarity and heterogeneity of CHDs), retrospective study designs, lack of multicentre and multidisciplinary collaboration, reliance on only standard fetal cardiac planes and still images, a variable focus on distinguishing normal from abnormal hearts or on identifying specific CHD types and limited investigation into the clinical utility.^20^

In the CAIFE study, we aim to address these limitations by close multidisciplinary collaboration between clinicians and engineers. Our goal is to establish an extensive well-annotated database of ultrasound scans from normal and CHD-affected fetal hearts, collected from multicountry sites. This database will be used for future development, training and validation of AI models for fetal echocardiography that use image and video analysis, enabling support for clinicians in the prenatal detection of fetal CHDs. This initiative is particularly critical in nonspecialist settings and LMICs, where expertise in fetal echocardiography is limited, and holds the potential to reduce global neonatal morbidity and mortality.

## Methods and analysis

### Study design

#### Study arms

**Figure 1:**
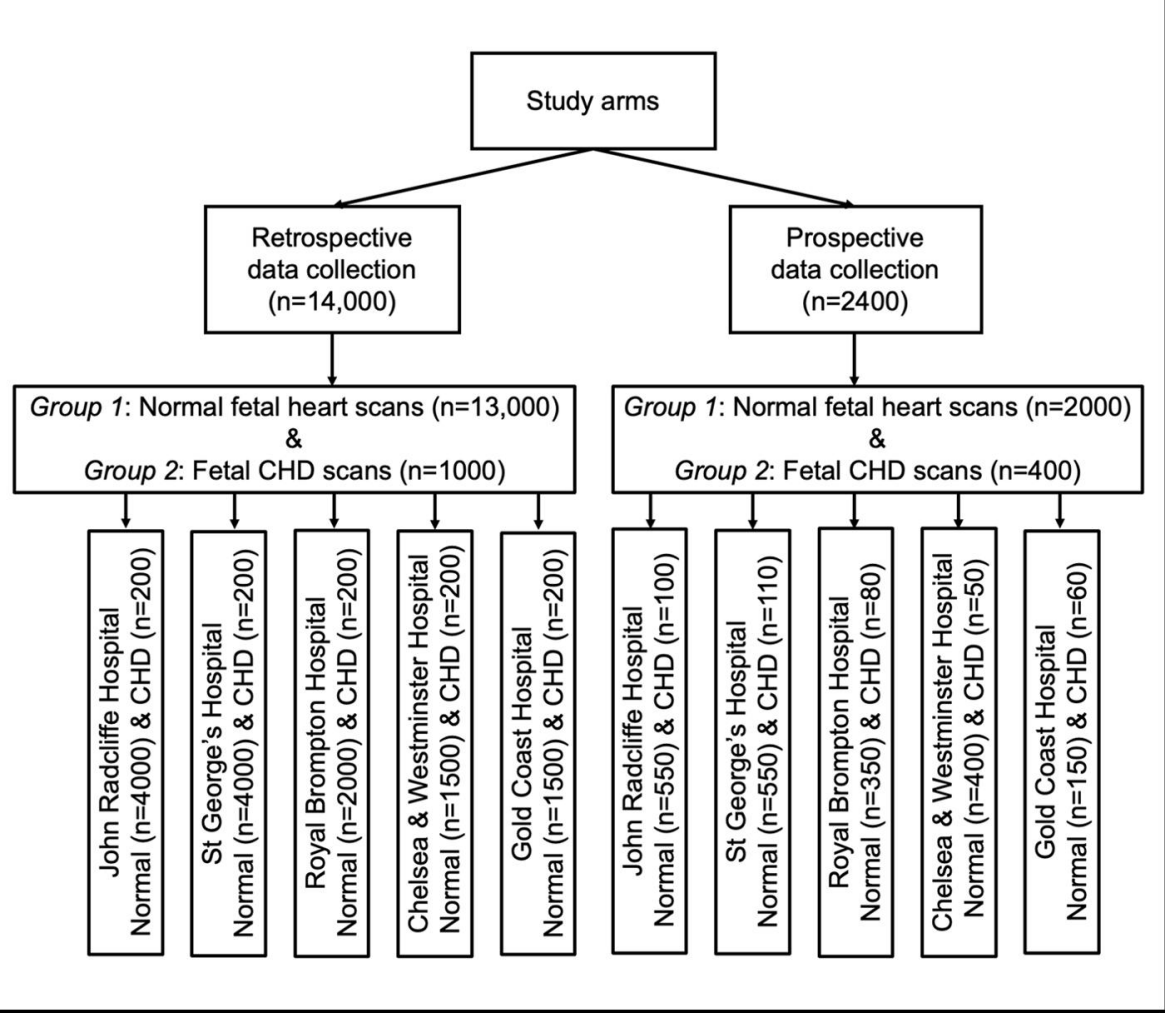
Study design. The diagram demonstrates the study arms, study groups and required data collection from participating hospital sites. CHD, congenital heart defect.

The study includes two data collection arms: retrospective and prospective (figure 1). Data will be collected in both arms from two types of ultrasound scans: echocardiograms performed by fetal cardiologists and obstetric ultrasound scans performed by sonographers or obstetric doctors.

#### Study groups

There are two study groups:

Group 1: Pregnant women whose fetuses have normal hearts, defined as negative for structural and functional heart disease and fetal arrhythmia.

Group 2: Pregnant women whose fetuses have major CHDs, as confirmed by clinical reports and visual verification of the CHD lesions by clinical experts (figure 1).

#### Study sites

The Clinical Artificial Intelligence Fetal Echocardiography (CAIFE) project is a multicentre international study that includes five hospital sites for data collection: (1) John Radcliffe Hospital, Oxford University Hospitals NHS Foundation Trust, UK; (2) Royal Brompton and Harefield Hospitals, Guy’s and St Thomas' NHS Foundation Trust, London, UK; (3) St George’s University Hospitals NHS Foundation Trust, London, UK; (4) Chelsea and Westminster Hospital NHS Foundation Trust, London, UK and (5) Gold Coast University Hospital, Southport, Australia (figure 1).

#### Start and end dates of the study

The study started on 7 September 2023. The planned end date of the study is on 30 November 2025.

### Participant identification

#### Retrospective study

Retrospective data will come from women who previously attended one of the collaborating hospitals for an obstetric ultrasound scan or a fetal cardiology appointment where they received a fetal cardiac scan. Ultrasound scans will be selected from two groups: pregnant women with uncomplicated pregnancies whose fetuses have a normal heart (n=13 000) and pregnant women whose fetuses have been diagnosed with a CHD (n=1000). The targets for retrospective data collection at each hospital site are shown in figure 1.

#### Prospective study

Participants will include pregnant women attending an obstetric ultrasound scan or fetal cardiology appointment in the Obstetric Ultrasound Departments or Fetal Cardiology Centre at the collaborating hospitals. Ultrasound scans will be undertaken in two groups: pregnant women with uncomplicated pregnancies whose fetuses have a normal heart (n=2000) and pregnant women whose fetuses have been diagnosed with CHDs (n=400). The targets for prospective data collection at each hospital site are shown in figure 1.

### Inclusion and exclusion criteria

#### Inclusion criteria

For the retrospective study, the inclusion criteria are any ultrasound scan that includes fetal heart data and where associated maternal clinical data available.

For the prospective study, the inclusion criteria are pregnant women attending an antenatal ultrasound scan or fetal cardiology appointment, aged 18 years or older, willing and able to give informed written consent and able to understand the study information provided. Both those with normal fetal hearts (defined as negative for structural and functional heart disease, fetal arrhythmia, chromosomal/genetic abnormalities and noncardiac congenital malformations) and those diagnosed with a CHD (by review of the clinical report and visual verification of ultrasound by a fetal cardiologist) are eligible.

#### Exclusion criteria

The exclusion criteria are any significant maternal or fetal disease or disorder which, in the opinion of the clinician, may put the participant at risk, negatively influence the study outcome or impair the ability to participate. Additionally, subjects with minor fetal cardiac abnormalities, such as minor valve abnormalities, mild valvular regurgitation, mild ventricular disproportion, small pericardial effusion, aberrant right subclavian artery and other normal variants will be excluded.

### List of collected CHDs

The following is a comprehensive list of 52 CHDs (with abbreviations) to be included in the study and is listed alphabetically:

AAI—aortic arch interruption, APVS—absent pulmonary valve syndrome, APW—aortopulmonary window, AS—aortic stenosis, CAT—common arterial trunk or truncus arteriosus, CAVSD/B—complete atrioventricular septal defect with balanced ventricles, CAVSD/U—complete atrioventricular septal defect with unbalanced ventricles, CCHD/LVOTO—any complex CHD with left ventricular outflow tract (LVOT) obstruction, CCHD/RVOTO—any complex CHD with right ventricular outflow tract (RVOT) obstruction, CC-TGA—congenitally corrected transposition of the great arteries, CM—cardiomegaly, COA—coarctation of the aorta, CT—cardiac tumours, DAA—double aortic arch, DAC—ductus arteriosus constriction, DCM—dilated cardiomyopathy, DILV/NRA—double inlet left ventricle with normally related great arteries (NRA), DILV/TGA—double inlet left ventricle with transposed great arteries (TGA), DORV/NRA—double outlet right ventricle with normally NRA (NRA-TOF type), DORV/SS—double outlet right ventricle with side-by-side great arteries (SS type), DORV/TGA—double outlet right ventricle with transposed great arteries (TGA type), DTV—dysplastic tricuspid valve, EA—Ebstein anomaly, FA/B—fetal arrhythmias/bradycardia, FA/T—fetal arrhythmias/tachycardia, FOR—foramen ovale restriction, HCM—hypertrophic cardiomyopathy, HLHS—hypoplastic left heart syndrome, ILA—isolated left isomerism, LSVC—left superior vena cava, MA/VSD—mitral atresia with ventricular septal defect, PA/IVS—pulmonary atresia with the intact interventricular septum, PA/VSD—pulmonary atresia with a ventricular septal defect, PAPVC—partial anomalous pulmonary venous connections, PAVSD—partial atrioventricular septal defect, PE—pericardial effusion, PS—pulmonary stenosis, RAA—right aortic arch, SI—situs inversus, TAPVC/C—total anomalous pulmonary venous connections (cardiac type), TAPVC/IC—total anomalous pulmonary venous connections (infracardiac type), TAPVC/SC—total anomalous pulmonary venous connections (supracardiac type), TAVSD/NRA—tricuspid atresia with ventricular septal defects and normally NRA, TAVSD/TGA—tricuspid atresia with ventricular septal defects and transposed great arteries (TGA), TGA/IVS—complete transposition of the great arteries with intact interventricular septum (simple TGA), TGA/VSD—complete transposition of the great arteries with ventricular septal defect (VSD) or other CHDs, TOF/DAA—tetralogy of Fallot with double aortic arch, TOF/LAA—Tetralogy of Fallot with left aortic arch, TOF/RAA—tetralogy of Fallot with right aortic arch, UVH/LAI—univentricular heart with left atrial isomerism, UVH/RAI—univentricular heart with right atrial isomerism, VSD—ventricular septal defects.

### Study procedures

#### Retrospective study

**Figure 2:**
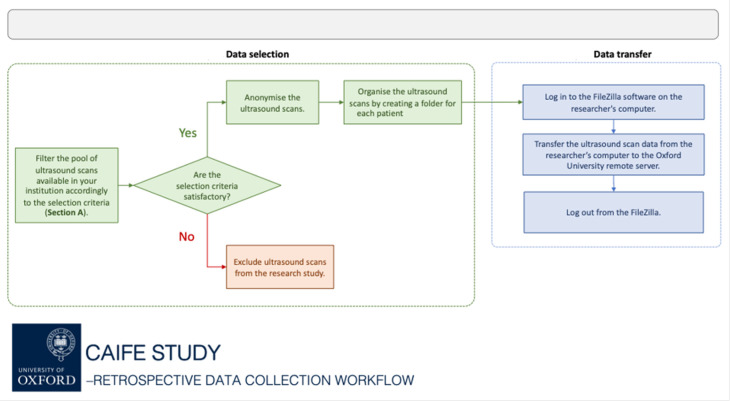
Retrospective data collection flow chart. The flow chart shows two steps of retrospective data collection: (1) data selection and (2) data transfer.

We will collect retrospective fetal ultrasound scans from structurally and functionally normal hearts and from those with CHDs (figure 2). The ultrasound scans and related clinical information will be the data routinely collected as part of the clinical service of participating hospitals. Given the retrospective nature, there is no direct participant involvement. Instead, hospital sites, with appropriate ethical and management approvals for the use of anonymised data, including the UK National Data Opt-Out system, will pool the data for analysis at the University of Oxford. To do this, ultrasound scans will be identified by the local hospital site teams. Ultrasound scans of the fetus and fetal heart will be retrieved by the approved local staff from the hospital sites from storage platforms (MEDCOM, ViewPoint, Agfa software). Associated clinical data will be collected from their electronic patient records (EPRs). All data are then anonymised before being shared with the research team and transferred securely to the University of Oxford.

#### Prospective study

**Figure 3:**
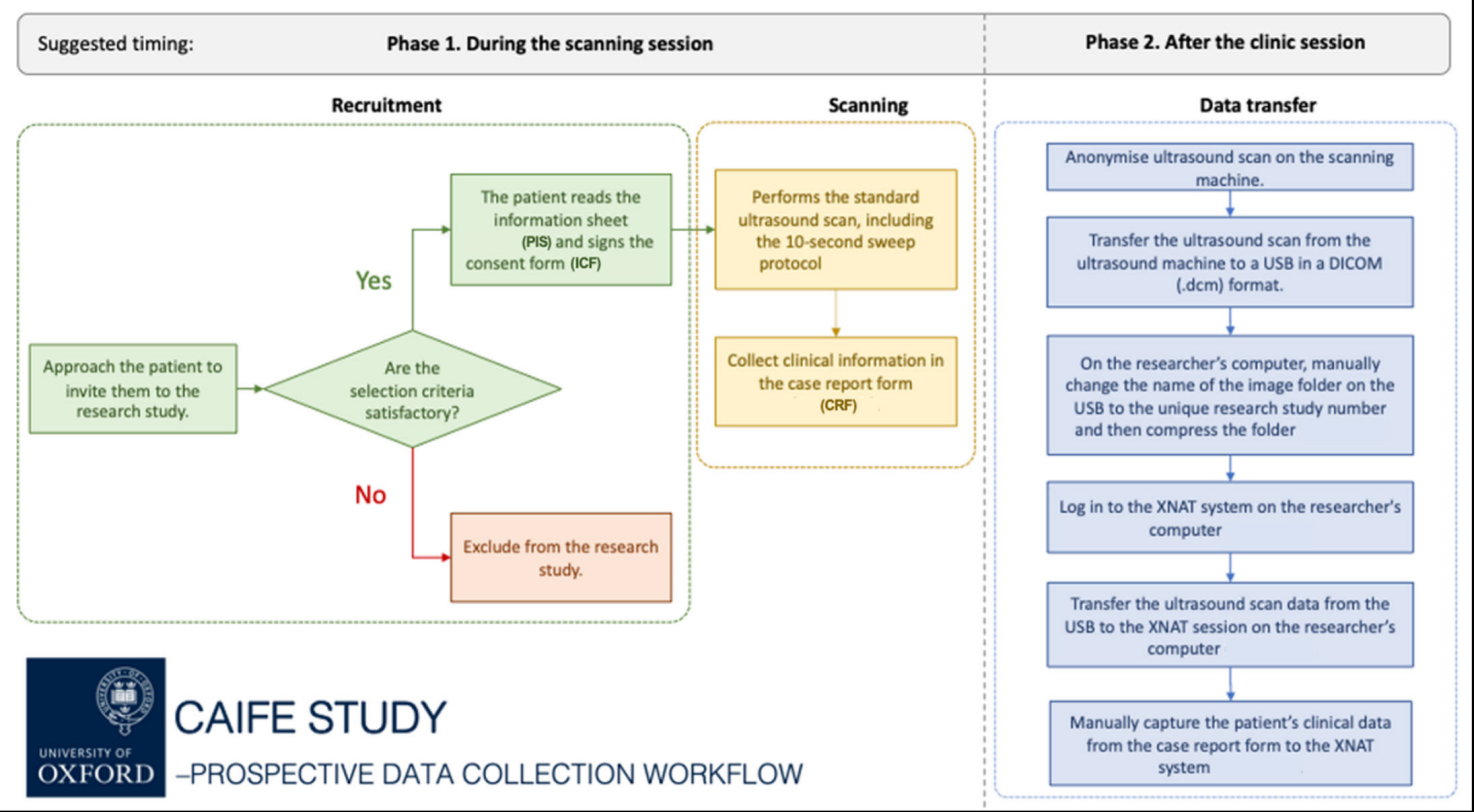
Prospective data collection flow chart. The flow chart shows two phases of prospective data collection: Phase 1: during the scanning session (recruitment and scanning) and Phase 2: after the clinical session (data transfer).

**Figure 4:**
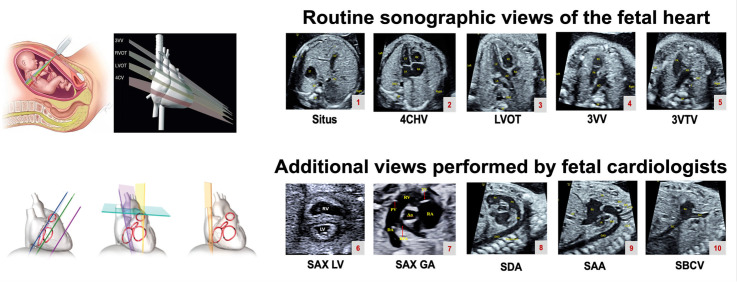
Fetal cardiac image planes for retrospective and prospective data collection. The figure shows cardiac scanning planes from routine obstetric (cardiac views 1–5) and fetal cardiology (cardiac views 1–10) scans which will be collected retrospectively and prospectively for the CAIFE study. 4CHV, four-chamber view; 3VTV, three-vessel trachea view; 3VV, three-vessel view; LVOT, left ventricular outflow tract; SAA, sagittal aortic arch; SAX GA, short axis great arteries; SAX LV, short axis left ventricle; SBCV, sagittal bicaval view; SDA, sagittal ductus arteriosus.

**Figure 5:**
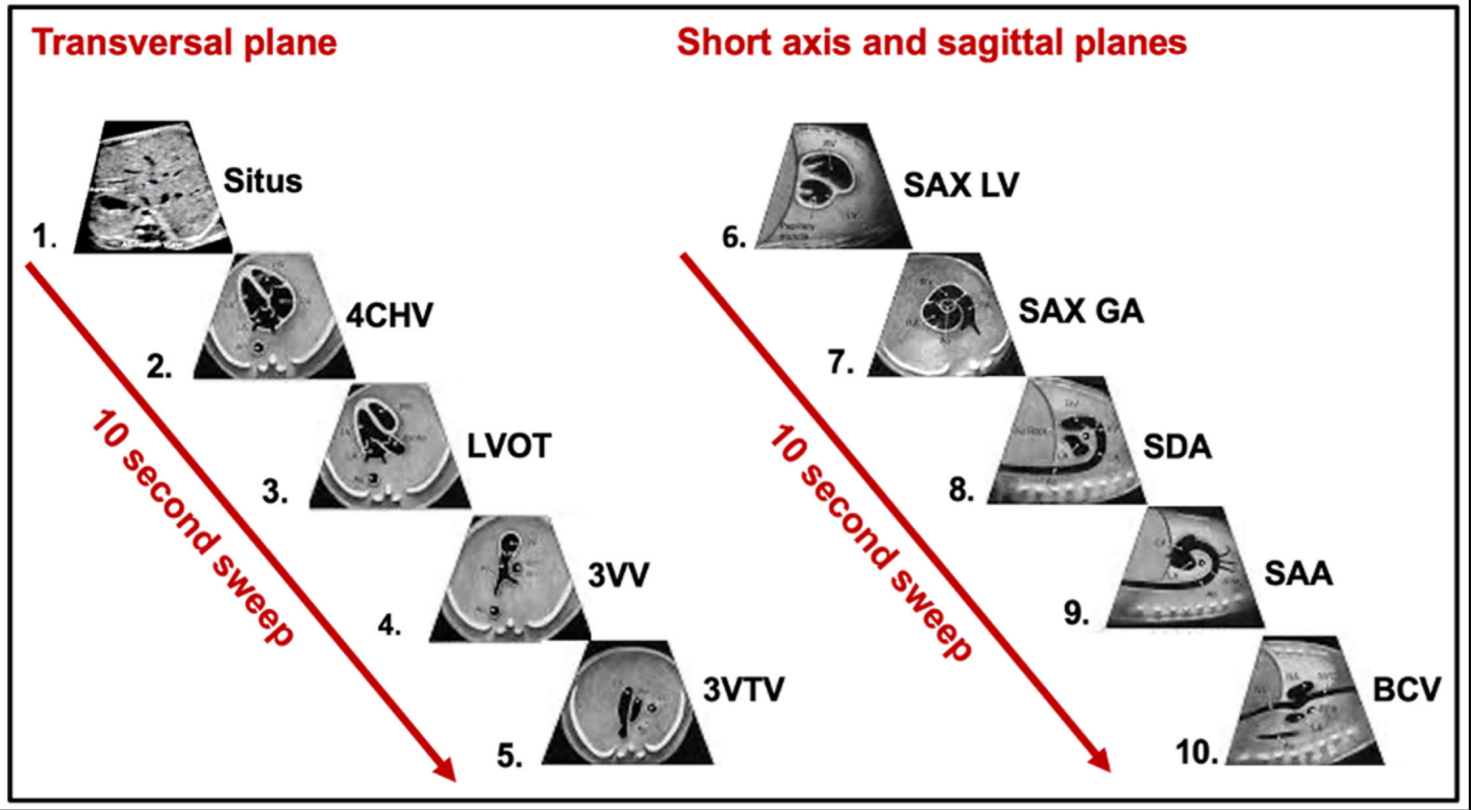
Prospective CAIFE 10 s sweeps. The figure shows the research-specific CAIFE 10 s sweeps in the transversal plane [a CAIFE T-sweep] (*sequences from 1 to 5*) and short axis/sagittal plane (a CAIFE S-sweep) (*sequences from 6 to 10*) for collecting fetal heart ultrasound data prospectively. 4CHV, four-chamber view; 3VTV, three-vessel trachea view; 3VV, three-vessel view; BCV, bicaval view; LVOT, left ventricular outflow tract; SAA, sagittal aortic arch view; SAX GA, short axis of the great arteries; SAX LV, short axis of the left ventricle; SDA, sagittal ductal view.

We aim to prospectively collect ultrasound datasets and associated clinical data from pregnant women attending routine second- and third-trimester obstetric or fetal cardiology scans, covering gestational ages from 16 to 40 weeks (figure 3). The data collection will include standard ultrasound images acquired during the scheduled appointment (figure 4) as well as 10 s long research-specific CAIFE sweeps of the fetal heart (figure 5) from fetuses with normal hearts and those diagnosed with CHDs. Women whose fetuses are not diagnosed with CHDs will be recruited during routine ultrasound examinations or through the fetal cardiology unit if scans initially suspected CHDs but were later determined to be normal. Women whose fetuses are diagnosed with CHDs will be recruited during their specialist scan with a fetal cardiologist. Once a potential participant is identified, they will be approached by a research team member to discuss the study. After providing informed consent, the routine scan will be performed, followed by a minimum of one 10 s CAIFE sweep of the fetal heart in several ultrasound planes. An informative scan is defined as one in which all fetal heart structures are visualised. Due to the complexity of capturing comprehensive information in a single sweep, sonographers may perform up to five CAIFE sweeps in each plane to gather the necessary data. All data received from participating sites will have appropriate approvals in place, including ethics approvals, as well as data-sharing contracts.

#### Eligibility assessment

All pregnant women attending antenatal ultrasound scans are potentially eligible, regardless of medical history. Participants must satisfy all the inclusion and exclusion criteria outlined in the study protocol.

#### Informed consent

With the appropriate permissions in place, women attending scheduled ultrasound scans will be approached by the research or trained clinical team member to ask if they would like to consider participating in the study and provided with the Participant Information Sheet. Research staff will explain the study in detail, highlighting the exact nature of the study and its implications, that there are no known risks and that participation is entirely voluntary. Women will have the opportunity to ask questions and take time to decide. If willing to participate, they will be asked to complete a written informed consent form (ICF). A participant is free to withdraw from the study without prejudice to future care, without affecting legal rights and with no obligation to give the reason for withdrawal. If needed, for example, if a woman would like more time to consider, arrangements will be made for further discussion and scans at a convenient time to minimise the participant’s burden.

#### Study Visits

For consented participants, the research study scan will be embedded within their routine scan and conducted using standard departmental ultrasound machines. In addition, at least one 10 s long CAIFE sweep of the fetal heart will be acquired (figure 5). The research scan will add approximately 10 min to the appointment. Clinical data, including maternal weight, maternal height, gestational age at the scan and the clinical fetal cardiac diagnosis will also be recorded from the woman’s medical records. Following the scan, participants will continue with routine pregnancy care. Participants may contribute additional scans at later gestational ages if approached, and consent is obtained.

#### Discontinuation/withdrawal of participants from study

Participants can withdraw from the study at any time, and their data will be removed if requested. The participants may also be withdrawn for reasons including ineligibility identified post-enrolment or withdrawal of consent. The reason for withdrawal will be recorded in the case report form (CRF), but participants are under no obligation to give a reason for withdrawal.

#### Definition of end of study

The end of the study will be once 14 000 retrospective and 2400 prospective fetal ultrasound scans have been collected.

## Data collection and annotation

### Retrospective and prospective data collection

Retrospective and prospective data collection will include fetal cardiac still images and short videos from the routine clinical obstetric care scans^24 25^ and fetal cardiology scans^26^ which include some of the following standardised views: cardiac situs view (Situs); four-chamber view (4CHV); left ventricular outflow tract (LVOT); three-vessel view (3VV); three-vessel trachea view (3VTV); short axis view of the left ventricle (SAX LV); short axis view of the great arteries (SAX GA); sagittal view of the ductal arch (SDA); sagittal view of the aortic arch (SAA) and sagittal bicaval view (SBV) (figure 4). We aim to collect recordings of pulsed wave Doppler signals across all fetal cardiac valves and routine clinical measurements of chambers, valves and ventricular walls obtained retrospectively and prospectively during the fetal cardiology scan (aortic and pulmonary valve end-systolic dimensions; mitral and tricuspid valve end-diastolic dimension; left and right ventricular end-diastolic width and length; and left and right atrial end-systolic width and length) (online supplemental figures S1 and S2). Where data are available, we will gather additional clinical measurements of chambers, valves and ventricular walls (online supplemental figure S3).

### Prospective CAIFE sweeps acquisition

Additionally for prospective data collection, research-specific CAIFE 10 s sweeps will be captured where fetal cardiac images are acquired by moving the ultrasound probe in a sequential, consistent and controlled manner through the fetal heart. Two CAIFE sweeps will be performed: T-sweep (in the transversal plane) and S-sweep (in the short axis/sagittal planes). The scanning trajectory of the T-sweep includes the five standard obstetric sonographic views in the transversal plane of the fetal heart.^27^ It starts from the Situs, followed by the 4CHV, LVOT, 3VV and 3VTV (figure 5, *sequences 1–5*). The scanning trajectory of the S-sweep includes five standard views of the fetal heart in short axis/sagittal planes. It starts from the SAX LV, through the SAX GA, SDA, SAA, to the bicaval view (figure 5, *sequence 6–10*). Both T- and S-CAIFE sweeps were obtained using two-dimensional (2D) and then colour Doppler techniques, and also in the dual (split image) mode with 2D and colour Doppler CAIFE sweep on the same image. The sweep acquisition time is up to 10 s.

### Prospective CAIFE sweeps annotation

#### Definition of frame types in CAIFE sweeps for AI-based model training

**Figure 6:**
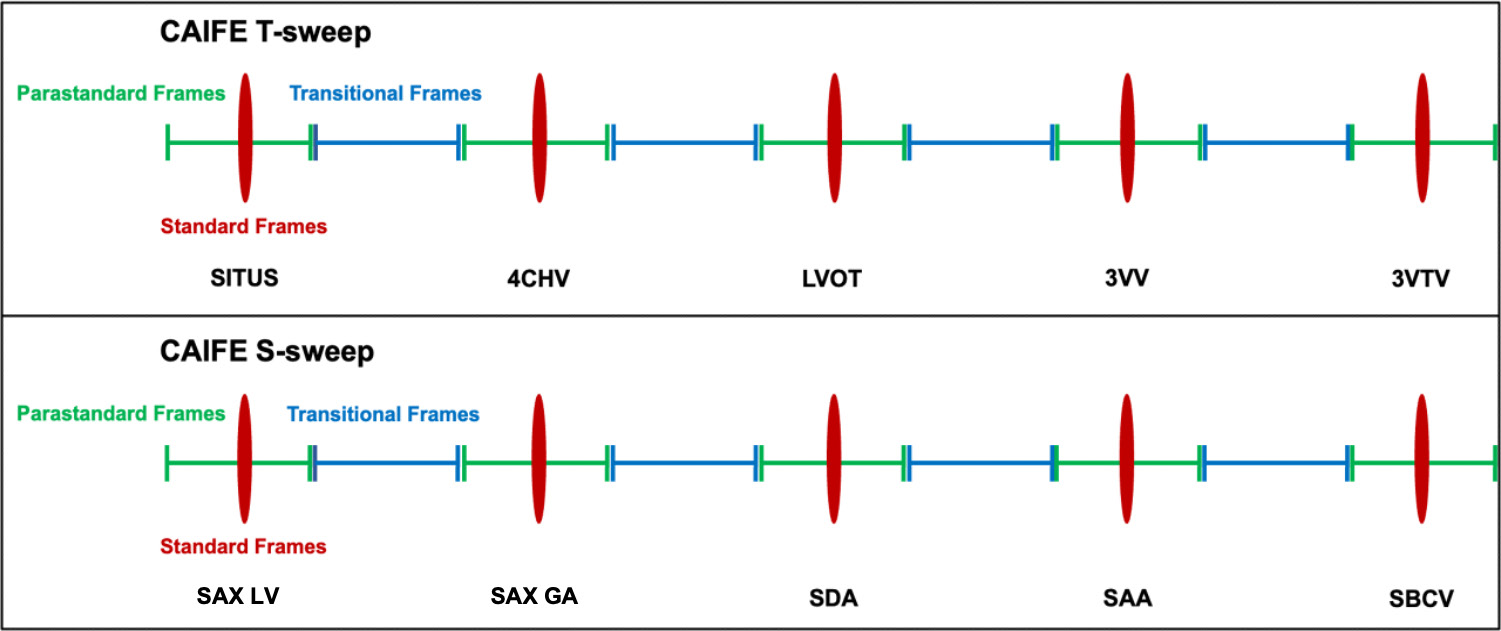
CAIFE sweep frames for AI training. Schematic Illustration of the standard, parastandard and transitional frames in relation to the standard sonographic or fetal cardiology views during acquisition of the T- and S-CAIFE sweeps. CAIFE, Clinical Artificial Intelligence Fetal Echocardiography; 4CHV, four-chamber view; 3VTV, three-vessel trachea view; 3VV, three-vessel view; LVOT, left ventricular outflow tract; SAA, sagittal aortic arch view; SAX GA, short axis of the great arteries; SAX LV, short axis of the left ventricle; SBCV, saggital bicaval view; SDA, sagittal ductal view.

Each of the 10 s CAIFE sweeps captures cardiac image frames containing a different amount of information relevant to the fetal heart examination. For the purpose of AI model training, we have defined three types of CAIFE sweep frames: standard, parastandard and transitional frames (figure 6).

##### Standard frames

In the transversal sweep (T-sweep), standard frames correspond to the ultrasound images displaying the heart structures according to the ISUOG Practice Guidelines^24^ (figure 5*, sequences 1–5*). In the short axis/sagittal sweep (S-sweep), these frames correspond to ultrasound images displaying the heart structures according to the guidelines outlined by the American Heart Association^26^ (figure 5*, sequences 6–10*).

##### Para-standard frames

In both T- and S- sweeps, these are ultrasound images acquired before or after the standard images where most of the heart structures can be identified but they are off-axis in relation to the standard view.

##### Transitional frames

In both T- and S-sweeps, transitional frames are ultrasound image frames containing fetal cardiovascular anatomy captured during the transition between two consecutive para-standard images.

### Description of normal fetal heart structures in CAIFE sweeps for AI model training

For the purpose of AI-based model development and training, we described the anatomical structures of the normal fetal heart visualised in the standard, parastandard and transitional frames of the CAIFE T-sweep (table 1) and S-sweep (table 2).

**Table 1:** Description of normal fetal heart findings in standard, parastandard and transitional frames of the CAIFE sweep in transversal plane

Image and frame description			
CAIFE T-sweep	Standard frames	Parastandard frames	Transitional frames
**Situs**	Determining fetal laterality from position of the fetus in utero The stomach is identified on the fetal left side of the fetal chest DAo is to the left side of the spine IVC is to the right side of the spine A short segment of the UV is seen	All above features are still visualised but off-axis from standard frames	DV is visualised Coronary sinus appearance (presence and size) Coronary arteries could be seen in some fetuses IVS and IAS integrity
**4CHV**	The heart sits in the left chest with a leftward apex (cardio-apex angle normal range=27°–67°) The heart comprises one-third of the thoracic cavity area (cardio-thoracic area normal range=0.42–0.50) Morphology of ventricles is identified Normal equal MV and TV end-diastolic dimensions (maximal width) Normal equal MV and TV size Normal laminar flow across MV and TV The integrity of IAS and IVS The FO flap bows into LA At least two pulmonary veins entering the left atrium Normal gap between the left atrium and DAo No pericardial effusion Normal appearance of myocardium Normal thickness of ventricular walls and IVS Normal cardiac rhythm and rate Normal ventricular function	All above features are still visualised but off-axis from standard frames	Muscular and perimembranous IVS integrity All pulmonary vein visualisation Myocardial appearance visualisation Coronary arteries could be seen in some fetuses
**LVOT**	Aorta (not bifurcating vessel) arises from LV Normal IVS—AV continuity (no VSD, aortic override) Normal AV—thin, opens well, not visible in systole No aortic stenosis/regurgitation/LV outflow tract obstruction Normal appearance of myocardium Normal ventricular function	All above features are still visualised but off-axis from standard frames All pulmonary vein visualisation	Normal crossing of the great arteries (pulmonary artery if anteriorly and to the right of the aorta)
**3VV**	Number of the vessels: three vessels—PA, Ao and SVC—no additional vessels, no absent vessels Position of the vessels (PA—on the left, then aorta and SVC on the right) Size (PA>Ao> SVC) Course (PA and DA to the left side of the chest) Flow in the vessels—the same direction in PA and Ao (no flow reversal) Normal PV (thin, opens well, not visible in systole) No pulmonary stenosis/ pulmonary regurgitation/RV outflow tract obstruction	All above features are still visualised but off-axis from standard frames PA divides into two branches: left and right branch pulmonary arteries Confluent normal equal size PA branches with normal flow	Normal continuity of the aorta to the ductus arteriosus Normal size of the transversal aortic arch Normal course of the transversal aortic arch Normal flow in the transversal aortic arch Normal appearance, size and flow in the ductus arteriosus
**3VTV**	Number (three vessels—PA, Ao and SVC—plus trachea, no additional vessels, no absent vessels) Position (PA is on the left, then aorta, crossing from right to left and SVC on the right, trachea is between spine and SVC) Size (PA=Ao or PA slightly>Ao> SVC; trachea=SVC) Course (PA and Ao are in front of the trachea running to the left chest forming ‘V-shape’ in front of the trachea) Flow across PA and aorta is in the same direction (no flow reversal) Size of Ao isthmus and DA and their ratio (>0.64)	All above features are still visualised but off-axis from standard frames Presence/size of thymus Normal branching of the aorta (aberrant right subclavian artery) Innominate (brachiocephalic vein) course and size	Normal branching of the aorta (aberrant right subclavian artery) Innominate (brachiocephalic vein) course and size Presence/size of thymus

Ao, aorta; AV, aortic valve; CAIFE, Clinical Artificial Intelligence Fetal Echocardiography; DA, ductus arteriosus; DAo, descending aorta; DV, ductus venosus; FO, foramen ovale; IAS, interatrial septum; IVC, inferior vena cava; IVS, interventricular septum; LV, left ventricle; LVOT, left ventricular outflow tract; MV, mitral valve; PA, pulmonary artery; PV, pulmonary valve; RV, right ventricle; SVC, superior vena cava; TV, tricuspid valve; UV, umbilical vein; VSD, ventricular septal defect; 3VTV, three-vessel trachea view; 3VV, three-vessel view.

**Table 2:** Description of normal fetal heart findings in standard, parastandard and transitional frames of the CAIFE sweep in the short axis/sagittal plane

Image and frame description			
CAIFE S-sweep	Standard frames	Parastandard frames	Transitional frames
**SAX LV**	An even size of the ventricles Normal global systolic function Normal appearance of myocardium Structurally and functionally normal mitral valve (no evidence of MV cleft) Intact IVS	All above features are still visualised but off-axis from standard frames	Normal connection of the aorta to LV No IVS aortic override Normal connection of the pulmonary artery to RV
**SAX GA**	Normal AV structure (three fine cusps open well) Normal PV (thin, opens well, not visible in systole) Normal AV and PV size and AV/PV ratio No pulmonary stenosis/pulmonary regurgitation/RV outflow tract obstruction No tricuspid regurgitation	All above features are still visualised but off-axis from standard frames	No flow reversal in the ductus arteriosus
**SDA**	Normal forward flow in the ductus arteriosus No flow reversal in DA No evidence of ductus arteriosus constriction/tortuosity/aneurysmal dilatation by 2D, colour Doppler and pulsed wave Doppler No evidence of azygous vein as a continuation of an interrupted IVC	All above features are still visualised but off-axis from standard frames	No flow reversal in the ductus arteriosus or aortic arch No evidence of azygous vein as a continuation of an interrupted IVC
**SAA**	Normal continuity of the aortic arch Normal and even aortic arch size Normal forward flow in the aortic arch No flow reversal in aortic arch No evidence of coarctation shelf No evidence of azygous vein as a continuation of an interrupted IVC Normal aortic arch branching pattern	All above features are still visualised but off-axis from standard frames	No flow reversal in the ductus arteriosus or aortic arch No evidence of azygous vein as a continuation of an interrupted IVC
**SBCV**	Normal connections of IVC and SVC draining to the right atrium Normal IVC course visualised from the diaphragm to the right atrium Normal size of IVC and SVC Normal DV insertion and flow pattern Normal flow in IVC and SVC	All above features are still visualised but off-axis from standard frames	N/A

AV, aortic valve; CAIFE, Clinical Artificial Intelligence Fetal Echocardiography; DV, ductus venosus; GA, great arteries; IVC, inferior vena cava; IVS, interventricular septum; LV, left ventricle; MV, mitral valve; N/A, not applicable; PV, pulmonary valve; SAA, sagittal aortic arch view; SAX, short axis view; SBCV, sagittal bicaval view; SDA, sagittal ductus arteriosus view; SVC, superior vena cava.

## Statistics and analysis

### Statistical methods

We will compare computational model outcome against the clinical diagnosis by calculating the true positives, true negatives, false positives and false negatives rates. For the purposes of detecting structures, we will compute results using statistical metrics, namely, sensitivity, specificity and accuracy. This includes calculating positive and negative predictive values for each outcome, compared with manual annotation.

For measurement agreement, we will compute Bland-Altman plots, intraclass correlation coefficients and the Dice coefficient, as for previous large-scale ultrasound agreement studies.^23 28^ This analysis will include transforming measurement data to Z-scores to account for the variability of increasing fetal size with gestation.^29^ The data analysis will be done simultaneously with the data collection.

### Sample size calculation

Building an accurate AI model requires large datasets.^30^ We undertook an extensive literature search to estimate the amount of data required. Three related recent works^3^,^5^ on fetal echocardiographic image deep learning-based modelling have used the following number of ultrasound videos to train and test models:

1. Dataset used in Truong *et al*^16^: A retrospective study where the study population was derived from a database of singleton 20-week fetuses; the proportion of CHD cases was 14.1%. The authors mentioned that the limitation of the study was a single-centre investigation, and thus selection bias by a population specific to the centre’s location might have been introduced. Thus, the work demonstrates the requirement for a large number of prospective ultrasound scans for successful deep learning-based models.

2. Dataset used in Arnaout *et al*^12^: A retrospective study with a training set of 1326 ultrasound scans from 18- to 24-week fetuses, and a test set included 4108 fetal ultrasound scans; the proportion of CHD cases was 0.9%. The authors noted that further testing of the model prospectively and in multiple centres, including community/nonexpert centres, would be required as the following work. They concluded that more training data from more centres may further boost performance and allow for the diagnosis of specific types of CHDs.

3. Dataset used in Han *et al*^31^: A retrospective study of 204 (2.1%) fetuses diagnosed with CHDs and 9453 (97.9%) fetuses with normal hearts. The researchers noted that the study sample size was small, and future studies would require a multicentre and large-sample investigation to validate the current findings.

In the CAIFE study, we plan to collect 14 000 retrospective ultrasound scans consisting of 13 000 normal fetuses and 1000 fetal ultrasound scans diagnosed with CHDs, as well as 2400 prospective ultrasound scans from pregnant women (2000 with normal fetal hearts and 400 fetuses with a CHD), for a total of 16 400 retrospective and prospective ultrasound scans from the participating sites.

## Study outcomes

### Primary outcomes

We aim to build up a large well-annotated database of ultrasound images of normal and abnormal fetal hearts for further development and training of AI-based models that are capable of detecting anatomical structures of both normal fetal hearts and fetal hearts with CHDs using cardiac ultrasound scans.

### Secondary outcomes

Additionally, clinical data will be collected to determine the effect of clinical parameters on AI-based model performance. These data will include maternal and fetal details, such as maternal weight and height, gestational age at the time of the scan and the clinical diagnosis of the fetal heart findings.

### Safety reporting

No adverse events are associated with the study. To date, no known risk is associated with exposure to ultrasound technology. The sonographers will be appropriately trained following standardised protocols/procedures.

## Data management

### Source data

The source documents for this study will be hospital records and ultrasound scans. CRF entries will be considered source data if the CRF is the site of the original recording (eg, there is no other written or electronic data record). All documents will be stored safely in confidential conditions. On all study-specific documents, other than the signed consent, the participant will be referred to by the study participant number/code, not by name.

### Data recording and record-Keeping

#### Retrospective data

The ultrasound scans will consist of Digital Imaging and Communications in Medicine standard files represented by the extension ‘.dcm’. These scans are stored locally on the hospital’s data management system. Before sending the required files to the University of Oxford research team, these scans will be deidentified by selecting this option on the ultrasound machine or digitally masking any patient data on images with a panel on the local storage software (eg, MEDCOM, ViewPoint and Agfa). The relevant clinical data will be extracted from the EPR. Each patient dataset will be assigned a unique number to both the ultrasound and clinical data to enable the University of Oxford research team to link the anonymised clinical data to the ultrasound scan. All the data will be anonymised, so there are no personal data within the dataset. The University of Oxford team will not be able to link the ultrasound or clinical data to the participant’s details as the link between the study code and the patient will be held at the hospital site providing the data. The data at hospital sites will be stored according to the local hospital policies before being securely transferred to the University of Oxford via the secured file transfer protocol using the XNAT or FileZilla software. The research team staff transferring the data from the participating institutions will have password access.

#### Prospective data

Patients will be given a unique identifier when recruited to the study, which will be used on their ultrasound scans and assigned to the CRF. Ultrasound scans, CRF and ICF will be stored securely on the local hospital infrastructure using the unique study participant number according to local policy. Ultrasound scans will then be transferred to the University of Oxford using a secure file transfer programme. The collaborators at hospital sites will be responsible for deleting unnecessary data from their computers once transferred. The clinical data will be entered into the database hosted on the University of Oxford servers. Data from all participants will be kept for 15 years after the end of the study for future research. It will only be made available for other research anonymously; the study ID will be overwritten before sharing.

## Quality assurance procedures

The study may be monitored or audited according to the approved protocol, Good Clinical Practice (GCP), relevant regulations and standard operating procedures.

## Protocol deviations

A study-related deviation is a departure from the ethically approved study protocol or other study document or process (eg, consent process or administration of study intervention) or GCP or any applicable regulatory requirements. Any deviations from the protocol will be documented in a protocol deviation form and filed in the study master file. In the event of the inadvertent receipt of identifiable data in the retrospective anonymised data, researchers will notify the University of Oxford Data.

### Patient and public involvement

We did not involve patients or the public in the design, the conduct or the reporting of our research.

## Ethics and dissemination

We will disseminate the findings through regional, national and international conferences and through peer-reviewed journals. The study was approved by the Health Research Authority, Care Research Wales and the Research Ethics Committee (Ref: 23/EM/0023; IRAS Project ID: 317510) on 8 March 2023. All collaborating hospitals have obtained the local trust research and development approvals.

## Significance and outlook

This project will provide a sizeable fetal echocardiographic database consisting of structurally and functionally normal fetal hearts and those diagnosed with major CHDs. We will use the collection of scans to train the AI-based models to characterise the anatomy of a normal fetal heart compared with those diagnosed with major CHDs. The CAIFE project will provide relevant findings on the impact of AI-based echocardiography for prenatal detection of fetal CHDs that may lead to significant changes in clinical practice and guidelines in the future. The approach is potentially translatable to the nonspecialist and low-resource setting where fetal echocardiography expertise is limited or nonexistent. Due to its interdisciplinary nature, this research is of interest to AI researchers, engineers, educators and clinicians working in fetal/paediatric cardiology, fetal medicine and global maternal and child health.

## Supplementary material

10.1136/bmjopen-2025-101263 online supplemental file 1
