# Supplementary material for: Prenatal detection of congenital heart defects using the deep learning-based image and video analysis: protocol for Clinical Artificial Intelligence in Fetal Echocardiography (CAIFE), an international multicentre multidisciplinary study
Source: BMJ Open. 2025 Jun 5;15(6):e101263. doi: 10.1136/bmjopen-2025-101263 (PMC12142171; doi:10.1136/bmjopen-2025-101263)
Supplement: online supplemental file 1 [file bmjopen-15-6-s001.pdf]

SUPPLEMENTAL DATA

Supplemental Figures

Figure S1. PW Doppler fetal cardiac measurements. Prospectively and retrospectively collected pulsed wave Doppler signal recording across all fetal cardiac valves obtained during a fetal cardiology scan. The figure shows pulsed wave Doppler traces across the aortic, pulmonary, mitral, and tricuspid valves obtained during a fetal cardiology scan.

Figure S2. **Fetal cardiac geometry measurements.** Two-dimensional routine fetal cardiac geometry measurements collected from prospective and retrospective fetal cardiology scans.

Figure S3. Additional fetal cardiac geometry measurements. Additional two-dimensional geometrical measurements collected prospectively and retrospectively from fetal cardiology scans.

SUPPLEMENTAL FIGURES

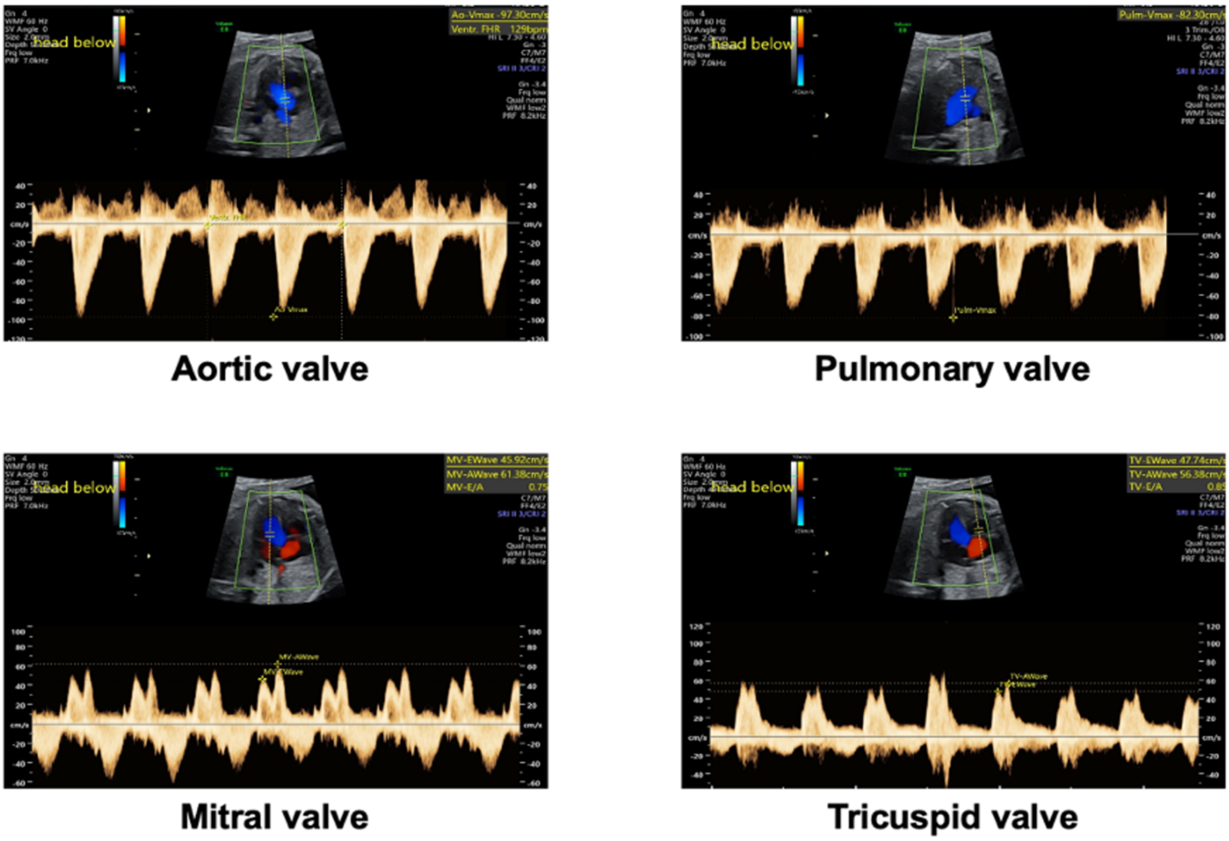

Figure S1. Prospectively and retrospectively collected pulsed wave Doppler signal recording across all fetal cardiac valves. The figure shows pulsed wave Doppler traces across the aortic, pulmonary, mitral, and tricuspid valves obtained during a fetal cardiology scan.

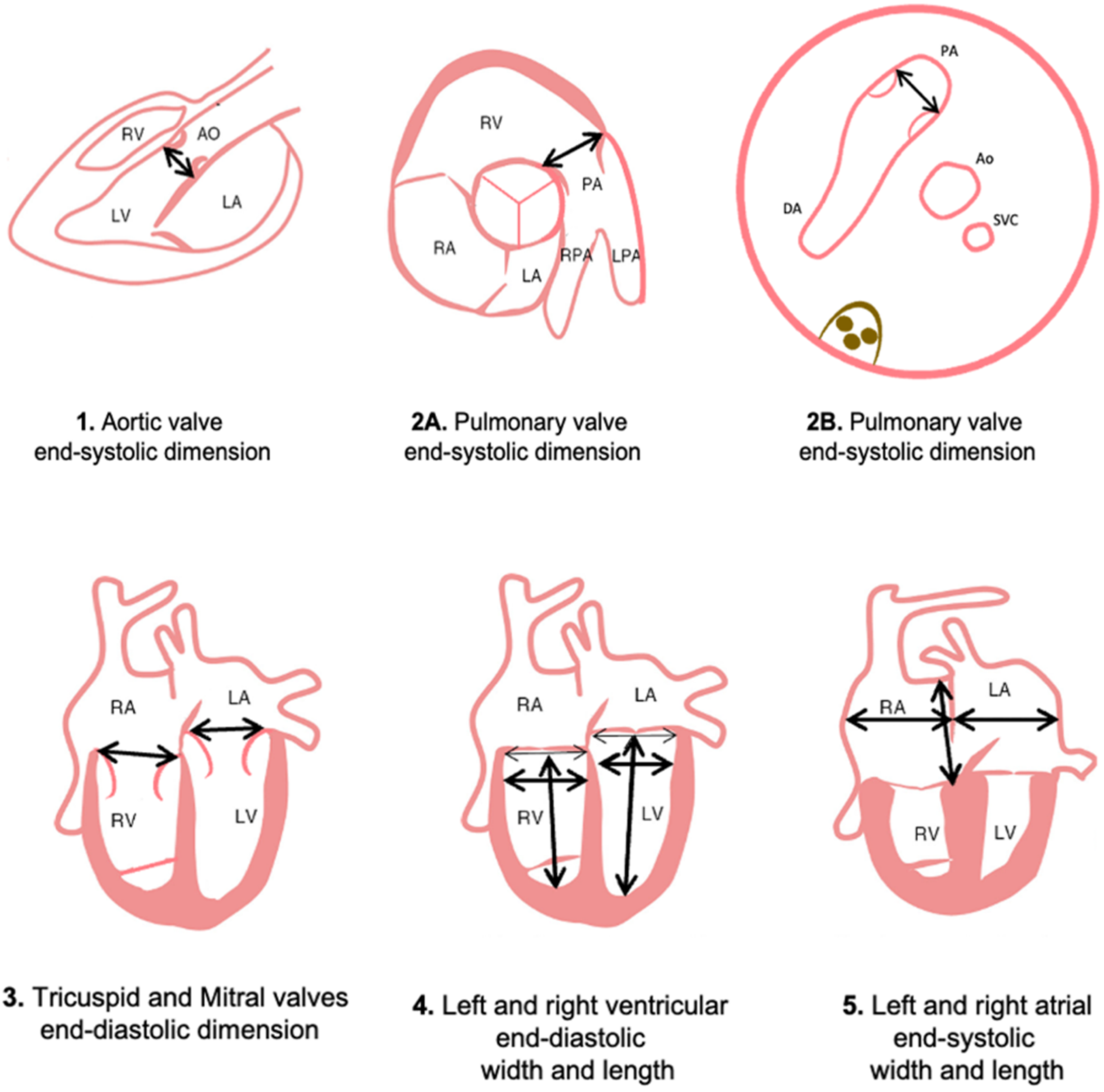

Figure S2. Fetal cardiac geometry measurements.

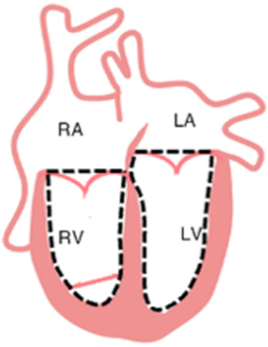

1. Left and right ventricular end-diastolic areas

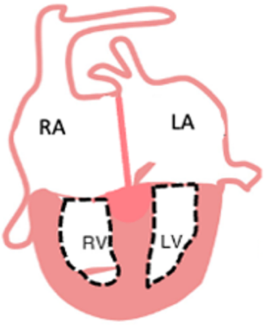

2. Left and right ventricular end-systolic areas

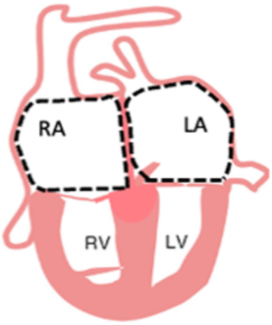

3. Left and right atrial end-systolic areas

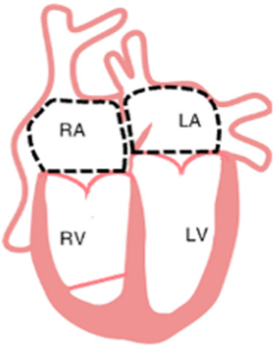

4. Left and right atrial end-diastolic areas

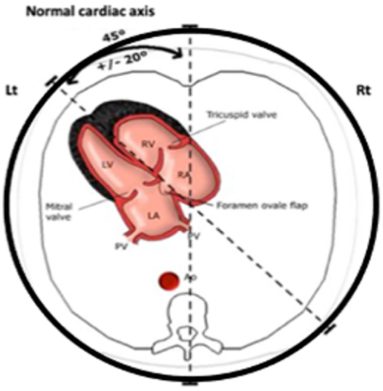

5. Cardiac axis angle

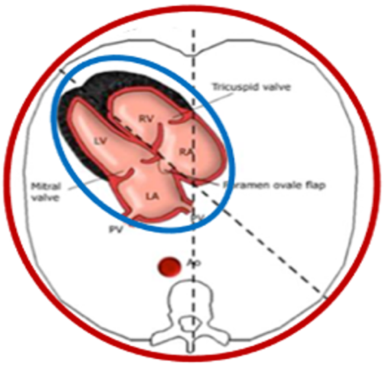

6. Cardio-thoracic area ratio

Figure S3. Additional fetal cardiac geometry measurements.
