# Supplementary material for: Prenatal detection of congenital heart defects using the deep learning-based image and video analysis: protocol for Clinical Artificial Intelligence in Fetal Echocardiography (CAIFE), an international multicentre multidisciplinary study
Source: BMJ Open. 2025 Jun 5;15(6):e101263. doi: 10.1136/bmjopen-2025-101263 (PMC12142171; doi:10.1136/bmjopen-2025-101263)
Supplement: Supplementary file 2 [file bmjopen-2025-101263.reviewer_comments.pdf]

PEER REVIEW HISTORY

BMJ Open publishes all reviews undertaken for accepted manuscripts. Reviewers are asked to complete a checklist review form and are provided with free text boxes to elaborate on their assessment. These free text comments are reproduced below.

ARTICLE DETAILS

Title (Provisional)

PRENATAL DETECTION OF CONGENITAL HEART DEFECTS USING THE DEEP LEARNING-BASED IMAGE AND VIDEO ANALYSIS: PROTOCOL FOR CLINICAL ARTIFICIAL INTELLIGENCE IN FETAL ECHOCARDIOGRAPHY (CAIFE), AN INTERNATIONAL MULTICENTRE MULTIDISCIPLINARY STUDY

Authors

Olga, Patey; Hernandez-Cruz, Netzahualcoyotl; D'Alberti, Elena; Salovic, Bojana; Noble , Alison; Papageorghiou, Aris T

VERSION 1 - REVIEW

**Reviewer**	**1**
**Name**	**Matsuoka, Ryu**
**Affiliation**	**Showa University - Hatanodai Campus, Ob & Gyn**
**Date**	**25-Mar-2025**
**COI**	**None**

This seems to be an important and significant protocol for AI social implementation of fetal cardiac ultrasound. I look forward to seeing it implemented and results obtained as soon as possible.

**Reviewer**	**2**
**Name**	**Day, Thomas G.**
**Affiliation**	**King's College London**
**Date**	**10-Apr-2025**
**COI**	**Co-founder and director of Fraiya Ltd, a spin-out company working in a similar area.**

This is a research study protocol for a mixed prospective and retrospective data collection exercise, with the ultimate aim being to train AI models to detect fetal CHD on ultrasound.

As the authors correctly point out, this is an important public health problem, as current detection rates are low despite well-developed screening programmes.

The protocol is detailed and well-written. I have the following comments:

Abstract:

- "CHD" should either be congenital heart disease, or congenital heart defects (plural).

- I'm not sure CHD is "rapidly emerging"- could the authors clarify what is meant by this statement?

Introduction:

- The definition of CHD is different to the abstract, and is now plural (CHDs- but disease is singular).

- "Of the 1.5 million children born with CHD each year, 96% live in low- and middle-income countries (LMICs)". This seems an extremely high figure, and I could not find evidence to support it in the supplied reference. Are the authors sure that this is correct?

Objectives and outcomes:

- These seem slightly muddled. Primary outcome usually refers to the outcome measure of a trial, not an "outcome objective". Also, this objective (to improve sensitivity and specificity" is reliant on the secondary outcome "objective" which is to train AI models.

- It would make more sense to me to have this protocol describe the data collection (and image annotation) aspects of the study, with the outcome being a large well-annotated database of ultrasound images of normal and abnormal fetal hearts.

- If the authors wish to include the AI models (either as an objective, an outcome, or in the statistical analysis section), then a lot more detail about these models is needed (training data, architecture, training methods, and model outcomes). Without this information, then I think relying on these models in terms of the objective of the study is confusing.

Other comments:

- Who will own the IP generated by this study (imaging data, trained model weights)? If the university, is there a plan to licence to any specific company?

- It might be worth clarifying about how consent will be gained for the retrospective data. If this has been waived by an ethics committee, will the NHS opt-out system be used? If not, how do the authors justify this?

VERSION 1 - AUTHOR RESPONSE

RESPONSES TO SPECIFIC COMMENTS OF REVIEWER 1:

Comments to the Author:
This seems to be an important and significant protocol for AI social implementation of

fetal cardiac ultrasound. I look forward to seeing it implemented and results obtained as soon as possible.
Response: The authors thank Dr. Ryu Matsuoka (Showa University, Hatanodai Campus) for the careful review of the manuscript, recognition the importance of our work and the positive comments.

RESPONSES TO SPECIFIC COMMENTS OF REVIEWER 2:

Comments to the Author:
This is a research study protocol for a mixed prospective and retrospective data collection exercise, with the ultimate aim being to train AI models to detect fetal CHD on ultrasound. As the authors correctly point out, this is an important public health problem, as current detection rates are low despite well-developed screening programmes.
The protocol is detailed and well-written.
Response: The authors thank Dr. Thomas G. Day (King's College London) for the careful review of the manuscript and the positive comments about our study.

I have the following comments:
Abstract:
- "CHD" should either be congenital heart disease, or congenital heart defects (plural).
Response: Thank you for this correction. We have clarified "CHD" abbreviation as singular and "CHDs" as plural one in both Abstract and Introduction *(Page 3, Lines 2-4; Page 5, Lines 2&5).*

- I'm not sure CHD is "rapidly emerging"- could the authors clarify what is meant by this statement?
Response: Thank you for this comment and allowing us to clarify. According to the global, regional, and national burden of congenital heart disease systematic analysis for the Global Burden of Disease Study 2017, the relative importance of CHD as a cause of child mortality is rapidly increasing, as evidenced by the increase in the proportion of total deaths due to CHDs for all (global, low, low-middle, middle, high-middle) social demographic index (SDI) countries, except for the high SDI countries (*GBD 2017 Congenital Heart Disease Collaborators* "*Global, regional, and national burden of congenital heart disease, 1990-2017: a systematic analysis for the Global Burden of Disease Study 2017*"*. Lancet Child Adolesc Health. 2020;4(3):185-200).* We have added this information to the manuscript *(Page 5, Lines 1-4).*

Introduction:
- The definition of CHD is different to the abstract, and is now plural (CHDs- but disease is singular).
Response: Thank you for this correction. We have clarified "CHD" abbreviation as singular and "CHDs" as plural one in both Abstract and Introduction *(Page 3, Lines 2-4; Page 5, Lines 2&4).*

- "Of the 1.5 million children born with CHD each year, 96% live in low- and middle-income countries (LMICs)". This seems an extremely high figure, and I could not find

evidence to support it in the supplied reference. Are the authors sure that this is correct?
Response: This information was taken from several publications: *GBD 2017 Congenital Heart Disease Collaborators. "Global, regional, and national burden of congenital heart disease, 1990-2017: a systematic analysis for the Global Burden of Disease Study 2017". Lancet Child Adolesc Health. 2020;4(3):185-200;*
Zühlke L, Lawrenson J, Comitis G, De Decker R, Brooks A, Fourie B, et al. "Congenital Heart Disease in Low- and Lower-Middle-Income Countries: Current Status and New Opportunities". Curr Cardiol Rep. 2019;21(12):163.
We added one more reference to strengthen this statement *(Page 5, Lines 6-7).*

Objectives and outcomes:
- These seem slightly muddled. Primary outcome usually refers to the outcome measure of a trial, not an "outcome objective". Also, this objective (to improve sensitivity and specificity" is reliant on the secondary outcome "objective" which is to train AI models.
-It would make more sense to me to have this protocol describe the data collection (and image annotation) aspects of the study, with the outcome being a large well-annotated database of ultrasound images of normal and abnormal fetal hearts.
Response: Thank you for these valuable comments. We agree with the Reviewer and have corrected the study outcomes according to the Reviewer's suggestions
(Page 6, Lines 2-6; Page 16, Lines 7-16).

- If the authors wish to include the AI models (either as an objective, an outcome, or in the statistical analysis section), then a lot more detail about these models is needed (training data, architecture, training methods, and model outcomes). Without this information, then I think relying on these models in terms of the objective of the study is confusing.
Response: Thank you for this valuable comment. We agree to leave the information about the AI model specifics out of this clinical protocol.

Other comments:
- Who will own the IP generated by this study (imaging data, trained model weights)? If the university, is there a plan to licence to any specific company?
Response: Thank you. The IP generated by this study will be owned by the University of Oxford. There is no current plan to licence it to any specific company.

- It might be worth clarifying about how consent will be gained for the retrospective data. If this has been waived by an ethics committee, will the NHS opt-out system be used? If not, how do the authors justify this?
Response: We thank the Reviewer for this important comment. The retrospective data collection was approved by the Ethic Committee *(Page 4, Lines 4-7).* As suggested, we added the information about the NHS Data Opt-Out system to the Methods *(Page 10, Lines 15-18).*
